# Performance evaluation of three rapid screening assays for detection of antibodies to hepatitis C virus in Cameroon

**DOI:** 10.1186/s13104-018-3465-8

**Published:** 2018-06-05

**Authors:** Clavel Landry Kouam Fondjo, Paul Alain Tagnouokam Ngoupo, Laure Ngono, Jean-Christophe Plantier, Richard Njouom

**Affiliations:** 1 Virology Department, Centre Pasteur of Cameroon, PO Box 1274 Yaounde, Cameroon; 2 Hôpital Charles Nicolle, Centre Hospitalier Universitaire de Rouen, 1 rue de Germont, Rouen, France

**Keywords:** Cameroon, HCV, Rapid diagnostic test, Performance, Plasma

## Abstract

**Objective:**

This study was aimed at evaluating the performance of three CE-marked rapid diagnostic tests (RDTs): Multisure-HCV, First Response^®^ and Toyo^®^; for screening anti- HCV antibody using plasma samples.

**Results:**

Overall, 200 plasma samples were used. Sensibility and specificity of these RDTs range from 71 to 99 and 78 to 100% respectively. Multisure scored a sensitivity at 99% (95% CI 97–100%) and First Response reached a specificity at 90% (95% CI 85–94.9%). Further studies should be conducted to establish an algorithm using these RTDs for the detection of HCV infection in Cameroon.

## Introduction

In Cameroon, the prevalence of hepatitis C virus (HCV) infection is estimated at 1.1% from the samples of the 2011 Demographic Health Survey [1]. This prevalence is estimated at 0.81% for the 15–49 year’s group and 2.51% for all individuals aged ≥ 15 years. It is estimated that about 195,000 individuals in Cameroon were viremic in 2011 including 92,000 adults aged 15–59 years and 103,000 individuals aged ≥ 60 years [1]. Diagnosis of HCV infection is based on the use of enzyme-linked immunoassays for the detection of HCV antibody followed by a molecular confirming test in case of positivity [2]. However, accessibility to these conventional assays is a challenge for the majority of people infected with HCV especially for those living in peripheral regions due to the high cost of these assays as well as their availability [3]. Diagnosis and testing remains a challenge for the elimination of viral hepatitis including HCV. According to WHO reports, only 1 in 5 people living with hepatitis C, were aware of their infection in 2015 [3]. Therefore, countries need to improve policies and programs to increase diagnosis. Rapid diagnostic tests (RDTs) represent an alternative solution to conventional HCV tests. Recently, certified RDTs for HCV have been approved to track down this infection [2]. The WHO prequalification program revealed that only two HCV RDTs (SD Bioline HCV from Standard Diagnostics and OraQuick HCV Rapid Antibody Test from OraSure Technologies) have been prequalified so far [4]. However, since 2014 in Europe, the French “Haute Autorité de Santé” (HAS) has evaluated CE-marked HCV RDTs and found that these tests had good performance. In addition, HAS recommends the use of CE-marked HCV RDTs that feature the European Union standard (100% of sensitivity and specificity ≥ 99%) [5, 6].

Data collected from the Ministry of Public Health in Cameroon showed that none of the HCV RDTs used in health facilities is qualified. Therefore this study aimed at evaluating the performances of three CE-marked HCV RDTs for the screening of anti-HCV antibodies using plasma samples collected in Resource Limited setting. These RTDs included: i) Multisure HCV Antibody Assay (MP Biomedical, Asia Pacific, Singapore), ii) First Response HCV Card Test (Premier Medical Corporation Ltd, Watchung, New Jersey), and iii) Toyo^®^ Anti HCV Test (Türklab, Izmir, Turkey).

## Main text

### Methods

#### Study sample and laboratory analysis

From November 2016 to February 2017, we carried out a cross-sectional study on 200 plasma (including 100 positive and 100 negative) stored at -80 °C at Centre Pasteur of Cameroon (CPC). These samples were previously screened for anti-HCV antibodies at the same laboratory using an automated chemiluminescent microparticle immunoassay (Architect anti-HCV assay; Abbott Diagnostics, Wiesbaden, Germany). Performance (sensitivity, specificity, positive predictive value and negative predictive value) of Multisure HCV Antibody Assay, First Response^®^ HCV Card Test, and Toyo^®^ Anti HCV Test were assessed for anti-HCV antibodies screening using plasma samples.

Multisure HCV Antibody is a qualitative immunochromatographic assay for the detection of antibodies to Core, NS3, NS4 and NS5 HCV proteins in human whole blood, plasma or serum. Each protein is revealed in a separate and distinct line in the test within 15 min. First Response HCV Card Test and Toyo^®^ Anti HCV Test are chromatographic immunoassays for qualitative detection of the antibodies against hepatitis C virus in human serum, plasma or whole blood samples. HCV antigens (Core, NS3, NS4 and NS5) are immobilized at only one test line and the result is obtained within 20 min.

The RDTs were performed following the manufacturer’s instructions, and the interpretation was done by three independent blinded laboratory technicians. No specific control was included in the study other than the internal control which is incorporated in each assay.

### Statistical analysis

Data was analyzed with SPSS statistics version 20.0.0 (IBM Corporation, USA). The relevant accuracy estimates of the three RDTs were expressed using the Pearson Chi square test, based on the result of Architect anti-HCV assay considered as Gold standard. The results were estimated with 95% confidence intervals (CI), and the difference was considered statistically significant with a p < 0.05.

### Results

We assessed the performance of Multisure HCV Antibody Assay, First Response^®^ HCV Card Test and Toyo^®^ Anti HCV Test in a total of 200; 140 and 150 plasma samples, respectively. The shortage in the remaining samples may explain the observed inequality in the number of RDTs used. The four parameters analyzed to assess the performances of these RDTs were the sensitivity (Se), the specificity (Sp), the positive predictive value (PPV), and the negative predictive value (NPV).

Multisure featured a clinical sensitivity of 99% (95% CI 97–100%) followed by First Response^®^ 96% (95% CI 92.7–99.2%) and Toyo^®^ 96% (95% CI 92.8–99.1%) (Table 1). As concerns the specificity, First Response^®^ scored 90% (95% CI 85–94.9%) followed by Multisure 83% (95% CI: 77.7–82.2%) and Toyo^®^ 78% (95% CI 71.3–84.6%) (Table 1). However, the sensitivity and the specificity were not significantly different among the three RDTs (p = 0.36 and p = 0.24 respectively). Regarding the predictive values, First Response^®^ scored the best PPV at 96% (95% CI 92.7–99.2%); meanwhile Multisure reached the best NPV at 99% (95% CI 97.6–100) (Table 1).

**Table 1:** Overall comparison of the three rapid diagnostic tests (RDTs) evaluated for the screening of anti-HCV antibodies

HCV RDTs	Se	Sp	PPV	NPV
	% (95% CI)	% (95% CI)	% (95% CI)	% (95% CI)
Multisure HCV Antibody assay	99 (97–100)	83 (77.7–88.2)	85 (80–89.9)	99 (97.6–100)
First Response^®^ HCV Card Test	96 (92.7–99.2)	90 (85–94.9)	96 (92.7–99.2)	90 (85–94.9)
Toyo^®^ Anti HCV Test	96 (92.8–99.1)	78 (71.3–84.6)	89.7 (83.9–94)	90.7 (86–95.3)

Se, sensibility; Sp, specificity; PPV, predictive positive value; NPV, negative predictive value; HCV, hepatitis C virus; RDTs, rapid diagnostic tests; CI, confidence interval

Since different serum samples were tested with each RDT, we assessed the performance of these tests with only the 90 overlapping samples to compare assay performance among all of the tests. Multisure had a sensitivity of 100% followed by First Response^®^ and Toyo^®^, 96% (95% CI 91.7–100%) (Table 2). As concerns the specificity, Multisure had a specificity of 92.5% (95% CI 87.5–97.4%) followed by First Response^®^ 90% (95% CI 84.7–95.2%) and Toyo^®^, 80% (95% CI 74.5–85.4%) (Table 2). However, the sensitivity and the specificity were not significantly different among the three RDTs (p = 0.35 and p = 0.20 respectively). Finally based on the ARCHITECT anti-HCV test results, we obtained 50/3 (true positive/false positive) and 0/37 (true negative/false negative) samples among the 90 overlapping samples with Multisure, 48/4 and 36/2 with First Response^®^ and 48/8 and 32/2 with Toyo^®^ (Table 2).

**Table 2:** Comparison of the three rapid diagnostic tests (RDTs) evaluated for screening anti-HCV with only overlapping samples

HCV RDTs	Se (95% CI)	Sp (95% CI)	PPV (95% CI)	NPV (95% CI)	TP (n)	FP (n)	TN (n)	FN (n)
Multisure HCV Antibody assay	100	92.5 (87.5–97.4)	94.3 (89.2–98.7)	100	50	3	37	0
First Response^®^ HCV Card Test	96 (91.7–100)	90 (84.7–95.2)	92.3 (87.3–97.2)	94.7 (90.1–99.3)	48	4	36	2
Toyo^®^ Anti HCV Test	96 (91.7–100)	80 (74.5–85.4)	85.7 (80.3–91.1)	94.1 (89.4–98.8)	48	8	32	2

*Se* Sensitivity, *Sp* specificity, *PPV* predictive positive value, *NPV* negative predictive value, *TP* true positive, *FP* false positive, *TN* true negative, *FN* false negative, *n* number, *HCV* hepatitis C virus, *RDTs* rapid diagnostic tests, *CI* confidence interval

### Discussion

This study showed none of the RDTs evaluated reached the European Union standards (100% of sensitivity and specificity ≥ 99%). These standards are based on different studies carried out in Europe, where conditions in realizing assays are practically different from those in resource limited countries in term of respect of quality assurance (supply chain reliability, implementation of a standardized logbook). The WHO (World Health Organization) has clearly demonstrated the high probability in obtaining false results using RDTs in case of non-respect of quality assurance [7]. Furthermore, the studied populations (North versus South) could also explain the difference observed in this study and European Union standards in term of specificity. Nevertheless, the results obtained from these RDTs are not significantly satisfactory and suggest that further studies should be conducted to establish an algorithm using these RTDs for the detection of HCV infection in Cameroon. The strategy used for HIV diagnosis based on two RDTs [8]: the most sensitive RDTs as the first, then the most specific assay in case of positivity, could be evaluated for HCV screening.

### Conclusion

None of the three RDTs evaluated met the European Union standards. However, the performances obtained are crucial indications for the Ministry of Public Health of Cameroon on the choice of RDTs to be used regarding their performance, and especially to promote assessment of HCV RDTs before implementation. Therefore, further studies should be conducted to establish an algorithm using these RTDs for the detection of HCV infection in Cameroon.

## Limitations

The evaluation of RDTs was only based on plasma. We did not consider that HCV serology can also be performed on the whole blood, serum and crevicular fluid. We could have worked with these four types of samples on the same panel to see if there are differences in the results.

## Abbreviations

DBS: dried blood spot
RDTs: rapid diagnostic tests
HCV: hepatitis C virus
HIV: human immunodeficiency virus
Se: sensitivity
Sp: specificity
PPV: positive predictive value
NPV: negative predictive value
